# Does psychopathology at admission predict the length of inpatient stay in psychiatry? Implications for financing psychiatric services

**DOI:** 10.1186/1471-244X-11-120

**Published:** 2011-07-29

**Authors:** Ingeborg Warnke, Wulf Rössler, Uwe Herwig

**Affiliations:** 1 Department of General and Social Psychiatry, Psychiatric University Hospital, Zurich, Switzerland

## Abstract

**Background:**

The debate on appropriate financing systems in inpatient psychiatry is ongoing. In this context, it is important to control resource use in terms of length of stay (LOS), which is the most costly factor in inpatient care and the one that can be influenced most easily. Previous studies have shown that psychiatric diagnoses provide only limited justification for explaining variation in LOS, and it has been suggested that measures such as psychopathology might be more appropriate to predict resource use. Therefore, we investigated the relationship between LOS and psychopathological syndromes or symptoms at admission as well as other characteristics such as sociodemographic and clinical variables.

**Methods:**

We considered routine medical data of patients admitted to the Psychiatric University Hospital Zurich in the years 2008 and 2009. Complete data on psychopathology at hospital admission were available in 3,220 inpatient episodes. A subsample of 2,939 inpatient episodes was considered in final statistical models, including psychopathology as well as complete datasets of further measures (e.g. sociodemographic, clinical, treatment-related and psychosocial variables). We used multivariate linear as well as logistic regression analysis with forward selection procedure to determine the predictors of LOS.

**Results:**

All but two syndrome scores (mania, hostility) were positively related to the length of stay. Final statistical models showed that syndromes or symptoms explained about 5% of the variation in length of stay. The inclusion of syndromes or symptoms as well as basic treatment variables and other factors led to an explained variation of up to 25%.

**Conclusions:**

Psychopathological syndromes and symptoms at admission and further characteristics only explained a small proportion of the length of inpatient stay. Thus, according to our sample, psychopathology might not be suitable as a primary indicator for estimating LOS and contingent costs. This might be considered in the development of future costing systems in psychiatry.

## Background

Industrialised countries are subject to high health care expenditure [1]. This particularly affects Switzerland, which is second in health costs after the US. In 2007, psychiatric hospitals spent about 2bn $ for mental health care, which is about 10% of all expenditure on inpatient treatment [2]. At present, length of stay (LOS) determines costs because hospitals are paid on a day to day basis. LOS is relatively long in Switzerland when compared to other industrialised countries [3]. In 2006, the average LOS in Swiss psychiatric hospitals was 44 days.

Due to high economic pressure, the introduction of new financing systems in inpatient psychiatry, such as prospective payment, is of public concern, not only in Switzerland but also in other countries. In somatic medicine, Diagnosis Related Groups (DRGs) have led to a reduction in LOS in several countries, including the US [4]. DRGs refer to patient groups that are clinically homogenous and that are associated with a fixed price for treatment [5]. Patients are grouped on the basis of variables that are commonly available from hospital discharge abstracts and that are assumed to have predictive power [6], (e.g. sociodemography, clinical characteristics and treatment). However, the field of psychiatry is still exempt from DRGs. In previous studies, psychiatric diagnosis could only explain up to 10-12% of the variation in LOS [7-11]. Accordingly, psychiatric inpatient treatment is usually still paid for on the basis of daily rates. The question thus arises whether other clinical measures could better predict LOS in inpatient psychiatry to find ways of modifying this cost-determining factor.

In view of the introduction of DRGs in Swiss somatic hospitals in 2012, several pilot projects are being conducted with respect to case-based (prospective) financing in the area of Swiss psychiatry [12,13]. In 2008, the Canton of Zurich started a project to investigate whether psychopathological syndromes according to the AMDP-system (referring to the working group on methods and documentation in psychiatry "Arbeitsgemeinschaft für Methodik und Dokumentation in der Psychiatrie") [14] assessed at hospital admission might be more appropriate than diagnosis for estimating resource consumption of psychiatric services. The major consideration was that the assessment of psychopathological syndromes is descriptive and free of theoretical considerations, whereas the validity of psychiatric diagnoses is questionable [15,16]. Assessing psychopathological symptoms is relatively easy for the trained psychiatrist and represents a clinical standard. Further, psychopathological syndromes are quantitatively measurable regarding their degree of expression, and thus dimensional. Psychopathological syndromes describe the status of a patient in a more sophisticated way and also consider pathology that does not yet lead to a diagnosis. Diagnostic categories are heterogeneous with regard to symptomatology and do not allow for a cumulative psychopathological effect [17,18]. Further, patients with a certain diagnosis such as schizophrenia may have very different psychopathology and social constraints, which may account for the resource consumption but is not considered in the diagnosis. Accordingly, some studies suggest that dimensional representations of psychopathology might be more appropriate for clinical practice than categorical ones [16,19].

Knowledge concerning the association between psychopathological syndromes or symptoms and LOS is limited. A recent pilot study showed that psychopathological syndromes at hospital admission explained less than 10% of the variation in length of stay in Swiss psychiatric inpatient care [20]. However, those findings have to be regarded as preliminary due to small sample size and limited analyses. Moreover, it remains unclear how much of the explained variation in LOS is due to psychopathological symptoms, the smallest entities of psychopathological measures, which were not investigated in the previous study.

Most of the previous studies that included diagnosis, sociodemographic and other patient-variables explained up to 20% of the variation of the LOS [21]. Variables that significantly increased the amount of explained variation or that were considered to be important determinants of LOS were for instance: type of admission [9], comorbidity, severity of illness [22] or level of functioning [22]. The amount of explained variation in LOS attained more than 20% in studies considering process-oriented variables like complications during hospitalisation and treatment factors [21,23].

The main objective of this study was to investigate whether psychopathology at admission (syndromes and symptoms) as assessed by the AMDP-system was suitable to predict the LOS by considering 3220 inpatient episodes. In this respect, our study adds on a previous investigation on syndromes and LOS that considered only a small sample [20]. Further, we considered other routinely collected variables that are usually mentioned as basic grouping criteria of DGRs or are cited as some of the most relevant predictor variables of LOS in the literature (e.g. sociodemography, treatment-related or further clinical data). We were primarily interested in variables assessed at hospital admission to obtain knowledge about the prognostic factors of resource use (in view of the discussion on prospective payment) but also considered treatment variables assessed during hospital stay. In principal, we were interested in finding implications for future financing of inpatient psychiatry.

## Methods

### Catchment area and central psychiatric register

Up to the year 2009, the catchment area of the Psychiatric University Hospital Zurich included approximately 350,000 inhabitants (today about 465,000). The hospital in question is one of six psychiatric institutions which serve a defined catchment area in the canton and which treat the whole spectrum of mental health problems. The Psychiatric University Hospital covers almost 40% of the treatment episodes of these hospitals. All Swiss cantons retrospectively collect patient data on sociodemographic variables, diagnosis according to the International Classification of Diseases (ICD-10) and treatment at hospital admission and/or discharge. Psychiatric hospitals of the Canton of Zurich cover additional information (e.g. data on psychopathology or severity of illness). The physicians in charge assess all medical data on the basis of a manual [13] and received special regular training in assessing psychopathology and functioning. The data were anonymised prior access to the study group. The ethical basis for the investigation, following the declaration of Helsinki, is given by the general permission of the legal responsible authorities. The collection of inpatient data in psychiatric hospitals was approved by federal law.

### Sample and data basis

Between 2008 and 2009 there were 5,224 hospital inpatient treatment episodes meeting specific inclusion criteria: Age 18 years and over and length of stay between 3 and 180 days. We were primarily interested in patients who entered the psychiatric hospital due to acute mental health problems with the need for treatment. Further, we assumed that hospital stays of more than 180 days would not necessarily be due to acute or chronic mental health problems but to other factors (e.g. social problems). We excluded admissions to the crisis intervention centre with obligatory hospital stay up to a maximum of 5 days. Complete data on AMDP were available in 3,220 of the 5,224 inpatient episodes, i.e. 62%. Complete data on psychopathology and additionally on further medical data were available in 2,939 (91%) of the 3,220 inpatient episodes.

We considered several potential predictor variables: Clinical factors were assessed at hospital admission and included several variables on psychopathology measured by the AMDP-system [14], which has been proven to be a valid instrument for this purpose [24]. It consists of 140 symptoms of different psychopathological domains (e.g. consciousness, fears and compulsions, affectivity, delusions, or somatic problems) and 9 syndrome scores. Examples of symptoms are "hopelessness" or "anxiety". The severity of each symptom was coded by 0 = no symptom/mild symptom severity, and 1 = moderate to strong symptom severity. The severity rating depends on the intensity and duration of symptoms [14]. In our statistical analyses, we considered only data with at least one available symptom out of all 140 symptoms. Nine syndrome scores according to the AMDP were derived by summing up specific symptom-scores: paranoid-hallucinatory syndrome, depressive syndrome, psycho-organic syndrome, manic syndrome, hostility syndrome, vegetative syndrome, apathy, compulsory syndrome, neurological syndrome. In total, those syndromes consist of 78 symptoms ranging in raw scores between 0 and 234. Apathy, for example, consists of 8 symptoms (cognitive inhibition, mental retardation, circumstantial thinking, narrowed thinking, low affectivity, affect rigidity, lethargy, social withdrawal). Summing up the respective severity ratings leads to a maximum syndrome score of 24. Syndrome data were left-skewed indicating that most of the patients had lower scores, whereas fewer patients had scores on the high end of the continuum. We considered syndrome scores but also split syndromes to group data around the median (lower syndrome score ≤ median vs. higher syndrome score > median; see below). Additionally, we used the GAF, which is a severity rating that assesses psychosocial functioning in daily life (e.g. work, social relationships). The GAF scores range between 0 (poor functioning) and 100 (very good functioning). We only considered GAF values with scores ≥ 1. The severity of illness (0 = not to moderately ill vs. 1 = markedly to extremely ill) was assessed by the Clinical Global Impressions Scale [25]. Finally, we considered the presence of a substance or personality disorder (main or secondary diagnosis; 0 = no vs. 1 = yes) because they comprise clinical and social aspects that are less susceptible for the AMDP.

We also took into account basic treatment-related variables assessed at hospital discharge. Categories were crisis intervention (action-oriented, limited time span, coping with acute crisis), psychotherapy (widely used, usually longer-lasting), integrated psychiatric treatment (clinical management with diverse approaches) and social interventions (management of daily activities). Additionally, we included acute care (treatment in an acute ward) as compared to specialised care, long-term care or care due to substance disorders. Finally, we considered compulsory treatment such as compulsory medical treatment or seclusion. The treatment-related variables were coded as dummy-variables (0 = no vs. 1 = yes). Sociodemographic variables included sex (0 = men, 1 = women), age (as a continuous variable), marital status (0 = widowed, divorced, separated, single vs. 1 = married), living situation (0 = in institution/homeless vs. 1 = own home) and employment status (0 = unemployed vs. 1 = employed). Finally, we considered admission-specific variables: way of referral (other = 1 vs. self = 1), legal basis of admission (0 = voluntary vs. 1 = compulsory), previous admission (0 = no, 1 = yes) and health insurance status (0 = private vs. 1 = general).

### Statistical analyses

We performed several descriptive sample comparisons: We did a drop-out analysis by comparing the sample of 3,220 patients finally included in statistical analyses with the sample excluded from analyses due to missing data on AMDP (N = 2,004) in terms of basic admission-specific patient characteristics (see table 1 also for applied statistical tests). Further, we compared the sample of N = 3,220 inpatient episodes with the sample that had complete data on several predictor variables besides psychopathology (N = 2,939) in terms of sociodemography and clinical variables.

**Table 1:** Characteristics of patients included in statistical analysis vs. those excluded

Variables	N (%) N = 2,004	N (%) N = 3,220	N (%) N = 5,224)
**Sociodemography**			

Age (Median, IQR)	44 (59-33) ****	43 (54-32) ****	43 (56-32)

Sex, male	1109 (55.3)	1697 (52.7)	2806 (62.3)

Marital status, married	351 (18.8)	603 (19.9)	954 (19.5)

Employment status, employed	488 (26.8)	873 (27.9)	1361 (27.5)

Living situation, own home	1068 (74.2)	1958 (72.1)	3026 (72.8)

**Clinical variables**			

Psychiatric disorder, only main diagnosis			

Organic disorder (ICD-10, F0), yes	182 (9.1) ****	190 (5.9) ****	372 (7.1)

Substance disorder (ICD-10, F1), yes	444 (22.2)	728 (22.6)	1172 (22.4)

Psychotic disorder (ICD-10, F2), yes	495 (24.7) ****	946 (29.4) ****	1441 (27.6)

Affective disorder (ICD-10, F3), yes	332 (16.6) **	642 (19.9) **	974 (18.6)

Anxiety disorder (ICD-10, F4), yes	165 (8.2) *	324 (10.1) *	489 (9.4)

Behavioural, psychosomatic disorders (ICD-10, F5), yes	6 (0.3)	8 (0.2)	14 (0.3)

Personality disorder (ICD-10, F6), yes	148 (7.4)	245 (7.6)	393 (7.5)

Severity of illness at admission, markedly to extremely ill	1446 (80.2) ****	2176 (70.4) ****	3622 (74.0)

Psychosocial Functioning (Median, IQR) ^§^	45 (55-35)	45 (56-33)	45 (55-33)

Severity of illness at discharge (improvement during hospital stay), unchanged to extremely worse	228 (13.2)	381 (12.8)	609 (12.9)

Length of stay ^†^	21 (42-9) *	23 (45-10) *	22 (44-10)

Abbreviations: IQR = interquartile range. * p < 0.05, ** p < 0.01, **** p < 0.0001.

Sample comparisons were done by the Chi-Square Test.

^† ^Sample comparison was done by the T-Test.

^§ ^Sample comparison was done by the nonparametric Mann Whitney U-Test.

Not all variables sum up to N = 3,220/N = 2,004 due to missing values as follows: 189/141 missings concerning marital status, 503/565 missings concerning living situation, 89/184 missings concerning employment status, 127/202 missings concerning severity of illness at admission, 270/1,264 missings concerning psychosocial functioning, 240/283 missings concerning severity of illness at discharge.

Preliminary analyses on predictors of the LOS were conducted by the Spearman correlation to analyse the relationship between discrete measures and the logarithmised LOS. We used phi-statistics to examine the association between dichotomised measures of psychopathology and binary LOS (≤ median vs. > median). Further, we compared characteristics of one final sample (N = 3,220) in terms of binary LOS (see table 2 also for applied statistical tests).

**Table 2:** Comparison of patients concerning LOS ≤ 23 days (median) vs. > 23 days (median)

Variables	LOS ≤ median (N = 1,629)	LOS > median (N = 1,591)	Total (N = 3,220)
**Sociodemography**			

Age (Median, IQR)	40 (50-31) ****	46 (60-34) ****	43 (54-32)

Sex, male	909 (55.8) ****	788 (49.5) ****	1697 (52.7)

Marital status, married	288 (18.7) ****	315 (21.1) ****	603 (19.9)

Employment status, employed	449 (28.2)	424 (27.6)	873 (27.9)

Living situation, own home	1004 (71.6)	954 (72.5)	1958 (72.1)

**Clinical variables**			

Psychopathological syndromes			

Paranoid-hallucinatory syndrome > 0 (median), yes	488 (30.0) ****	613 (38.5) ****	1101 (34.2)

Depressive syndrome > 4 (median), yes	678 (41.6) ****	815 (51.2) ****	1493 (46.4)

Psycho-organic syndrome > 0 (median), yes	418 (25.7) ****	528 (33.2) ****	946 (29.4)

Manic syndrome > 0 (median), yes	230 (14.1)	412 (25.9)	642 (19.9)

Hostility syndrome > 2 (median), yes	202 (12.4)	122 (7.7)	324 (10.1)

Vegetative syndrome > 0 (median), yes	341 (20.9) ****	420 (26.4) ****	761 (23.6)

Apathy > 3 (median), yes	653 (40.1) ****	846 (53.2) ****	1499 (46.6)

Compulsory syndrome > 0 (median), yes	74 (4.5)*	101 (6.3)*	175 (5.4)

Neurological syndrome > 0 (median), yes	106 (6.5) ***	153 (9.6) ***	259 (8.0)

Substance disorder (ICD-10, F1), yes	602 (36.9) ****	303 (19.0) ****	905 (28.1)

Personality disorder (ICD-10, F6), yes	214 (13.1) ****	121 (7.6) ****	335 (10.4)

Severity of illness at admission, markedly-extremely ill	984 (62.5) ****	1192 (78.5) ****	2176 (70.4)

Psychosocial Functioning (Median, IQR)	49 (60-35) ****	44 (55-30)****	45 (56-33)

Severity of illness at discharge (improvement during hospital stay), unchanged to extremely worse	283 (18.2) ****	98 (6.9) ****	381 (12.8)

**Admission-specific variables**			

Type of referral, self	456 (30.2) ****	311 (22.8) ****	767 (26.7)

Insurance type, public	1571 (96.4) ****	1483 (93.2) ****	3054 (94.8)

Compulsory admission, yes	554 (34.0)	505 (31.7)	1059 (32.9)

Previous admission, yes	457(28.1) **	374 (23.5) **	831 (25.8)

**Treatment variables**			

Compulsory medication, yes	65 (4.1) ****	110 (7.6) ****	175 (5.8)

Social seclusion, yes	70 (4.4) ****	111 (7.7) ****	181 (6.0)

Other compulsory interventions, yes	62 (3.9) ****	97 (6.7) ****	159 (5.3)

Crisis intervention, yes	842 (53.7) ****	293 (20.5) ****	1135 (37.9)

Psychotherapy, yes	64 (4.1) ****	167 (11.7) ****	231 (7.7)

Integrated treatment, yes	213 (13.6) ****	278 (19.5) ****	491 (16.4)

Advisory service	27 (1.7)	25 (1.8)	52 (1.6)

Abbreviations: LOS = length of stay. IQR = interquartile range. **** p < 0.0001, *** p < 0.001, ** p < 0.01 * p < 0.05.

Not all variables sum up to N = 3,220 due to missing values as follows: 189 missings concerning marital status, 503 missings concerning living situation, 89 missings concerning employment status, 127 missings concerning severity of illness at admission, 240 missings concerning severity of illness at discharge, 270 missings concerning psychosocial functioning, 342 missings concerning manner of referral, 197 missings concerning compulsory treatment, 225 missings concerning most important treatment.

For the final multivariate analyses, we only considered predictor variables which were significantly (p < 0.05) associated with each LOS variable in bivariate analyses. Regarding analyses of symptoms and LOS, we were only interested in those with correlations of at least r/r_φ _> (±) 0.100. Multivariate analyses on psychopathology were conducted by means of two statistical approaches: First, we used multiple linear regression analysis to investigate the amount of variation in LOS explained by the syndrome scores (sum of symptoms) or symptoms and further patient-characteristics as mentioned in section "sample and data basis". Second, we used multivariate logistic regression to find out about the odds ratios associated with each syndrome as a binary variable (≤ median vs. > median) using dichotomised LOS (LOS ≤ median vs. > LOS) as the dependent variable. In particular, R^2 ^from linear regression is more precise to identify the amount of variation in LOS whereas odds ratios from logistic regression are easier to interpret while describing the association between binary LOS and other factors. In each analysis, we used the forward selection procedure to find out which syndromes or symptoms best explained the LOS. Finally, we computed eight multivariate statistical models by using linear vs. logistic regression: Two models only included syndromes, two models only included symptoms and additional four models either covered syndromes or symptoms as well as sociodemographic, admission-specific and treatment-related variables. We did not include symptoms and syndromes in a single model because some of them were correlated (see results), which is due to the fact that some symptoms constitute specific syndromes.

The coefficient of determination, R^2 ^(corrected; linear regression)/Nagelkerke-R^2 ^(logistic regression), expresses the amount of explained variation. Concerning linear regression analysis, we back-transformed the regression-coefficient (B) and the 95% confidence interval (95% CI) from the log-scale to the original scale (EXP [B], EXP [95% CI]). Concerning logistic regression analysis, we showed effect coefficients and corresponding 95% CI (EXP [B], EXP [95% CI]). Statistical analyses were conducted by SPSS software [24].

## Results

### Demographic characteristics

The sample characteristics of the inpatient episodes finally included in statistical analyses are shown in tables 1 and 2. The median age was 43 years. 55% of the patients were males. A comparison of the final sample (N = 3,220) considered in multivariate analysis and the sample not included due to missing data on AMDP (N = 2,004) showed some differences: We had relatively less completed data from psycho-geriatric patients. The patients included compared to those not included were slightly younger, had a lower prevalence of an organic disorder, a higher prevalence of psychotic, affective or anxiety disorders and they were less severely ill at admission. Further, we found that patients included stayed slightly longer than patients excluded. Although this small difference was statistically significant it was not considered to be clinically relevant.

### Comparison with respect to LOS

About 49% of the patients in the final sample considered in multivariate analysis (N = 3,220) stayed longer than 23 days (median) in psychiatric hospital. A comparison of this sample concerning LOS (≤ 23 [median] vs. > 23 [median]) is provided in table 2.

### Predictors of the length of stay

**Figure 1:**
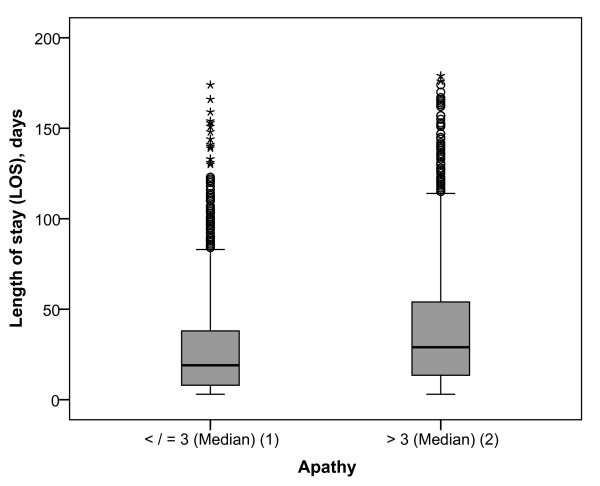
**Box-Plot of the length of stay mapped on logarithmised ordinate across the apathetic syndrome (≤ 3 [median] vs. > 3 [median])**. N = 3,220. (1): length of stay (LOS) = 19 days (median), interquartile range (IQR) = 30; apathy > 3 (2): LOS = 29 days (median), IQR = 41. Horizontal lines illustrate median and quartiles, vertical lines illustrate minimum and maximum of the LOS: (1): 3-171 days, (2): 3-176 days. Circles stand for outliers (values between 1.5 IQR's and 3 IQR's from the end of a box), asterisks stand for extreme values (more than 3 IQR's from the end of a box): (1): ≥ 84, (2) ≥ 115.

All but two syndrome scores (manic syndrome, hostility) positively correlated with the length of stay (LOS) in univariate analyses, regardless of the statistical approach used (Spearman vs. phi-statistics). The correlation coefficients ranged between r = 0.052/r_φ *= *_0.04 (compulsory syndrome) and r = 0.211/r_φ *= *_0.131 (apathy), indicating that higher syndrome scores were associated with longer LOS. A boxplot (Figure 1) provides an example of the association between apathy (≤ median vs. > median) and LOS mapped on a logarithmised ordinate.

For reasons of relevance, we only considered symptoms with correlation coefficients of r/r_φ _> (±) 0.100. Regarding the Spearman correlation, we found that 7 symptoms fulfilled this criterion: social withdrawal, morning depressiveness, disturbance of vitality, cognitive inhibition, anxiety, lethargy, ruminating. Regarding the phi correlation, 4 symptoms were "significantly" associated with the binary LOS-variable: social withdrawal, memory disturbance (short-term), morning depressiveness, memory disturbance (long-term). All these symptoms were positively related to LOS, which means higher scores were related to longer inpatient treatment. Most of the remaining symptoms with correlation coefficients ≤ r_φ *= *_0.100 were also positively related to LOS.

In the final statistical models conducted by linear regression, we did not consider the depressive syndrome because it was correlated with apathy (r = 0.501, N = 3,220; r = 0.503, N = 2,939), thus the respective results on apathy and LOS are comparable to those with the depressive syndrome and LOS (the latter results are not shown). Further, we did not include the GAF score because it was correlated with the CGI score (r = -0.504; N = 2,939). As mentioned above, we did not consider symptoms and syndromes in a single model because some of them were correlated as well. For example, we found correlations > r/r_φ _0.500, N = 3220; N = 2,939) between the depressive syndrome and the symptom disturbance of vitality or between apathy and the symptom social withdrawal.

We examined eight multivariate statistical models (tables 3 and 4). In the first model conducted by linear regression analysis and covering syndromes, three out of seven syndromes remained in the statistical model and explained 5% of the variation of the logarithmised LOS: paranoid-hallucinatory syndrome, apathy (associated with depressiveness) and psycho-organic syndrome (model 1). The logistic regression model on binary syndrome variables revealed that 5 syndromes led to an explained variation of at most 4% (model 2).

**Table 3:** Prediction of the length of stay of psychiatric inpatients by psychopathology (N = 3,220)

		Linear regression models				Logistic regression models ^†^		
	r **	**EXP (B)^+^**	**EXP (95% CI)^+^**	**Corr. R^2^**	r_φ **_	**EXP (B)**	**EXP (95% CI)**	**Nagelkerke R^2^**

**Syndrome scores**	**Model 1**				**Model 2**			

Intercept		16.22	15.29-17.09			0.564		

Apathy	0.211	1.04	1.04-1.06	0.040	0.131	1.39	1.19-1.62	0.023

Paranoid-hallucinatory syndrome	0.112	1.02	1.01-1.03	0.047	0.090	1.41	1.20-1.63	0.031

Psycho-organic syndrome	0.108	1.02	1.01-1.03	0.050	0.090	1.33	1.15-1.54	0.036

Depressive syndrome					0.096	1.36	1.16-1.59	0.042

Neurological syndrome					0.057	1.34	1.03-1.75	0.044

**Symptoms**	**Model 3**				**Model 4**			

Intercept		17.88	17.13-18.67			0.76		

Social withdrawal (apathy)	0.159	1.23	1.14-1.35	0.025	0.123	1.64	1.39-1.93	0.020

Memory disturbance (short-term) (psycho-organic syndrome)					0.104	1.43	1.10-1.86	0.033

Morning depressiveness (depressive syndrome)	0.130	1.57	1.33-1.85	0.036	0.110	2.71	1.82-3.98	0.044

Memory disturbance (long-term) (psycho-organic syndrome)					0.103	1.51	1.11-2.03	0.047

Disturbance of vitality (depressive syndrome)	0.101	1.16	1.06-1.27	0.043				

Cognitive inhibition (apathy)	0.128	1.25	1.09-1.45	0.047				

Anxious (other)	0.106	1.14	1.05-1.24	0.049				

Lethargy (apathy)	0.106	1.13	1.03-1.25	0.051				

Abbreviations: LOS = length of stay. ^+ ^EXP (B), EXP (95% CI): Back-transformed regression coefficient and 95% confidence interval from the logarithmised scale to the original scale; r = correlation coefficient (Spearman), r_φ _= correlation coefficient (phi statistics). ** p < 0.01.

^† ^LOS ≤ 23 days (median) vs. LOS > 23 days (median) was used as the dependent variable. In model 2, syndrome scores were split at the median (≤ median vs. > median): paranoid-hallucinatory syndrome > 0 (median) vs. ≤ median, yes; vegetative syndrome > 0 (median) vs. ≤ median; apathy > 3 (median) vs. ≤ median; compulsory syndrome > 0 (median) vs. ≤ median; neurological syndrome > 0 (median) vs. ≤ median; depressive syndrome > 4 (median) vs. ≤ median.

**Table 4:** Prediction of the length of stay by psychopathology and further characteristics (N = 2,939)

	Linear regression models			Logistic regression models ^†^		
	EXP (B) *	EXP (95% CI) *	**Corr. R^2^**	**EXP (B)**	**EXP (95% CI)**	**Nagelkerke R^2^**

**Syndrome scores & further characteristics**		**Model 5**			**Model 6**	

Intercept	21.08	18.33-24.23		0.925		

Crisis intervention, yes	0.53	0.49-0.56	0.158	0.29	0.24-0.34	0.152

Apathy	1.03	1.02-1.04	0.182			

Acute care, yes	0.73	0.67-0.78	0.202	0.47	0.39-0.58	0.176

Substance disorder, yes	0.78	0.72-0.83	0.219	0.53	0.44-0.64	0.203

Severity of illness, moderate to severe	1.27	1.19-1.37	0.233	1.72	1.43-2.06	0.221

Age	1.01	1.00-1.01	0.238	1.01	1.01-1.02	0.232

Psychotherapy, yes	1.27	1.12-1.43	0.241	1.83	1.32-2.53	0.237

Depressive syndrome				1.30	1.10-1.54	0.240

Compulsory medication, yes	1.33	1.16-1.53	0.244	1.64	1.15-2.33	0.243

Compulsory admission, yes	0.91	0.85-0.99	0.246			

Personality disorder				0.72	0.55-0.95	0.245

**Symptoms & further characteristics**		**Model 7**			**Model 8**	

Intercept	21.18	18.42-24.37		0.879		

Crisis intervention, yes	0.52	0.49-0.56	0.158	0.29	0.24-0.34	0.152

Acute care, yes	0.73	0.68-0.79	0.181	0.48	0.39-0.58	0.176

Substance disorder, yes	0.78	0.73-0.84	0.202	0.54	0.45-0.65	0.203

Severity of illness, moderate to severe	1.27	1.18-1.36	0.221	1.68	1.40-2.02	0.221

Social withdrawal (apathy), moderate to severe	1.17	1.08-1.26	0.232			

Age	1.00	1.00-1.01	0.237	1.02	1.01-1.02	0.232

Social withdrawal (apathy), moderate to severe				1.46	1.21-1.77	0.239

Disturbance of vitality (depressive syndrome), moderate to severe	1.13	1.04-1.23	0.241			

Psychotherapy, yes	1.25	1.22-1.41	0.244	1.85	1.34-2.55	0.244

Compulsory medication, yes	1.31	1.13-1.49	0.247			

Ruminating (depressive syndrome), moderate to severe	1.11	1.02-1.21	0.249			

Morning depressiveness (depressive syndrome), moderate to severe	1.21	1.03-1.42	0.250	1.83	1.17-2.86	0.247

Compulsory medication, yes				1.60	1.13-2.28	0.249

Lethargy (apathy), moderate to severe	1.09	1.01-1.21	0.251			

Personality disorder, yes	0.90	0.81-0.99	0.252	0.75	0.57-0.99	0.251

Abbreviations: LOS = length of stay. * EXP (B), EXP (95% CI): Back-transformed regression coefficient and 95% confidence interval from the logarithmised scale to the original scale.

^† ^LOS ≤ 23 days (median) vs. LOS > 23 days (median) was used as the dependent variable. In model 6, syndrome scores were split at the median (≤ median vs. > median): apathy > 3 (median) vs. ≤ median; depressive syndrome > 4 (median) vs. ≤ median.

Regarding linear regression analysis on symptoms, we found that 6 out of 7 symptoms explained 5% of the variation of the logarithmised LOS (model 3 in table 3). The corresponding model by logistic regression shows that 4 symptoms explained almost 5% of the variation of the LOS (model 4 in table 3). Depending on the statistical approach, social withdrawal increased the LOS by a factor of 1.2 to 1.6, and morning depressiveness increased the LOS by a factor of 1.6 to 3. We found that symptoms referring to apathy, the depressive or psycho-organic syndrome remained within the statistical models.

The final four models conducted by linear (models 5 and 7) or logistic regression analysis (models 6 and 8), including psychopathology as well as sociodemographic, admission-specific clinical and treatment-related characteristics, each resulted in an explained variation of about 25% (table 4). Other variables, as gender, were not significant and dropped out. With respect to psychopathology, only apathy (model 5) or the depressive syndrome (model 6) remained in the final statistical models. Further, symptoms that referred to apathy or to the depressive syndrome were finally included (models 7 and 8). As shown in table 4 admission for crisis intervention alone explained about 15% of the variation. Crisis intervention, acute care, substance abuse, compulsory admission (model 5) and personality disorder (models 6-8) were negatively related to LOS, whereas psychopathological syndromes (models 5 and 6) or symptoms (models 7 and 8), severity of illness (CGI) and psychotherapy were positively related. The depressive syndrome increased the LOS by a factor of 1.3 (model 6). Being more severely ill, receiving psychotherapy, the symptom "morning depressiveness" and compulsory medication increased the LOS by a factor of 1.6 to 1.8 (models 6 and 8). Patients who received crisis intervention, acute care or patients with a substance or personality disorder stayed 0.3-0.8 times less long than patients who did not receive such therapies or who did not have a substance disorder (models 6 and 8). In summary, the following variables were excluded from the final two linear regression models: sex, marital status, paranoid-hallucinatory syndrome, vegetative syndrome, compulsory syndrome, neurological syndrome, psycho-organic syndrome, insurance type, previous admission, integrated treatment, cognitive inhibition, anxiety. The following variables were excluded from the final two logistic regression models: sex, marital status, insurance type, type of referral, paranoid-hallucinatory syndrome, psycho-organic syndrome, vegetative syndrome, apathy, memory disturbance (short-term), memory disturbance (long-term), previous admission, integrated treatment.

## Discussion

The aim of our study was to analyse whether psychopathology as assessed by the AMDP-system at admission to psychiatric hospital as well as other variables (e.g. treatment assessed at the end of hospital stay) are suitable to predict LOS. The study was conducted in the context of the current discussion on new financing systems in Swiss psychiatry in order to gain knowledge bearing on future expenditure.

We examined eight multivariate statistical models. Psychiatric syndromes (models 1 and 2) or psychopathological symptoms (models 3 and 4) explained about 5% of the variation of LOS. The consideration of syndromes or symptoms and further characteristics (models 5-8) led to an explained variation of about 25%, with a weak association between AMDP-psychopathology and LOS. Apathy (model 5) or the depressive syndrome (model 6) were the only syndromes that remained in final statistical models. Further, symptoms that were included in model 7 or model 8 referred to the apathetic or depressive syndrome. Specific admission for crisis intervention explained about 15% of variation in LOS.

Our results enhance previous findings on the predictive power of syndromes with a smaller sample [20], as here psychopathological symptoms also do not allow sufficient prediction of LOS. Other clinical variables besides psychopathology such as substance abuse or severity of illness at admission had a minor influence on the length of stay as well, which is in line with previous findings taking several hospitals in a whole catchment area into account while controlling for the factor "hospital" [11]. According to a previous study [21,23], the consideration of variables related to treatment within hospital stay led to an explained variation of more than 20%.

One reason for the poor association between psychopathology at admission and the LOS could be attributable to its inherent characteristics. On the one hand, descriptive and dimensional measures of psychopathology might indeed better represent the patient's current mental condition than diagnosis as outlined in the introduction. However, the changeability of psychopathology implies that it could be affected by factors within and beyond inpatient treatment, which might influence LOS. Accordingly, changes in clinical condition might be better related to LOS than severity of illness at hospital admission. An earlier study reported that grouping patients on the basis of severity ratings that take the treatment process into account (e.g. symptoms from admission to discharge, level of care, response to therapy, acute symptoms at discharge) led to an explained variation of the LOS of up to 50% [26]. Regarding clinical practice, imagining a severely manic and/or psychotic patient with high psychopathological scores who is rapidly remitting under adequate medication and discharged after 10 days, also because he or she desires this, would be an example of high scores on psychopathology and a short stay. On the other hand a schizophrenic patient with low acute psychopathology but with a disturbed social network outside, for instance regarding appropriate accommodation, might long remain in hospital until the necessary subsequent support is initiated. Another example would be a patient with an acute but rapidly remitting depressive crisis versus a patient with a depressive personality and a complicated course of illness including social problems.

We further considered treatment-related variables within hospital stay or compulsory medication which were assessed at hospital discharge. However, usually physicians determine an appropriate treatment strategy right at the beginning of a patient's hospital stay, which could be adjusted over time in hospital. Obviously LOS is more strongly related to a specific global treatment approach (in this study crisis intervention or acute care) characterised by its duration compared to clinical measures, whenever these are less well-defined categorisations susceptible to subjective estimations. There might be further clinical or social factors associated with the patient's medical condition. This could refer to etiological features of the mental disorder as heredity, childhood or other trauma or psychosocial burden. Little is known about the relationship between psychosocial needs [27], chronicity of the mental illness or response to previous treatment [27] and the LOS. The variable social support has been considered as an important predictor of LOS in previous studies [28].

Further, there might be factors unrelated to the patient which influence LOS. For example, studies including organisational variables (e.g. number of staff, ward, type of hospital) show an explained variation of more than 20% [21]. The inclusion of variables referring to the care system (e.g. number of staff, contact rate in outpatient care, sociodemographic structure) also led to an explained variation of 20% [29]. It is not clear how much of the variation in LOS is due to factors like treatment philosophy of a hospital or the physician in question or further structural variables (e.g. waiting time before referral to another institution, quality of outpatient care). However, such "external" factors are not related to individual treatment needs. Nevertheless, the findings mentioned give important hints as to factors that influence LOS. Such results on predictors might facilitate the physician's appropriate assessment of LOS [6].

Our results might have implications for future research on LOS and payment in inpatient psychiatry. First, it might be worthwhile to focus on patients with higher apathy or depressive syndrome. There seems to be a need for investigating (or developing) clinical measures that are more strongly related to clinical practice. The consideration of more detailed information on treatment in routine assessment could be promising. With respect to financing, our findings suggest that psychopathology at admission is not suitable to serve as a basis for estimating resource use. Another question is whether resource use could be sufficiently predicted at all. Some alternative models to prospective costing are currently examined. One example is the development of a budgeting system on a day to day basis which takes patient-characteristics and treatment into account [12,30]. At present, the Canton of Zurich is investigating whether mixed financing (combining daily rates and case-based remuneration) might be effective in reducing LOS and in preventing early readmissions [12].

We have to consider some limitations. The included sample contained relatively less patients from the psycho-geriatric wards than the excluded sample but a slightly higher proportion of patients with an affective or psychotic disorder, whenever the proportion of diagnoses between both samples was still of a comparable magnitude. We consider this limitation to be a minor one, because the assessed question on psychopathology and LOS presumably does not depend on such small differences concerning case mix, all the more as LOS and diagnosis are not strongly related [11]. Such, our results are to be regarded as valid for a case mix as can be found in general psychiatric hospitals with adult psychiatric patients. Further, data on validity or reliability of the clinical ratings are not available. However, physicians did receive special training in performing these ratings and they were performed as well as possible in the routine clinical setting. We used LOS as a proxy for resource consumption but LOS is only one of several factors (e.g. amount of service provision per day) that lead to costs. Our approach to assessing treatment variables was on a relatively unspecific level and should be made more specific if intended for assessing resource consumption. Finally, AMDP-data are here only related to one specific hospital (and one specific catchment area) in the whole Canton of Zurich. To validate our findings, it might be considered performing such investigations in other countries or in different healthcare systems.

## Conclusions

Findings on appropriate clinical predictors of length of stay (LOS) with respect to financing inpatient psychiatry are limited. We investigated the relationship between psychopathological syndromes and symptoms assessed at psychiatric hospital admission and LOS. In our sample, we did not find AMDP-symptoms or AMDP-syndromes to be suitable for predicting LOS. Accordingly, this does not indicate that those factors might be an appropriate basis for psychiatric cost estimates. Further research is needed to find either variables that better predict the LOS of inpatient episodes or alternative methodological approaches that better explain resource consumption.

## Competing interests

The authors declare that they have no competing interests.

## Authors' contributions

IW, WR and UH conceived of the study and study design. WR strongly contributed to the idea of examining psychopathology as probable predictor variable of the LOS. UH participated in all parts of the manuscript and coordination. IW participated in study design, carried out the statistical analyses and drafted the manuscript. All authors worked on the manuscript with important intellectual content and approved the final manuscript.

## Pre-publication history

The pre-publication history for this paper can be accessed here:

http://www.biomedcentral.com/1471-244X/11/120/prepub
